# COVID‐19 transmission: economy‐boosting investment should target innovation in pandemic containment strategies to minimize restrictions of civil liberties

**DOI:** 10.1111/1462-2920.15263

**Published:** 2020-10-13

**Authors:** Kenneth Timmis

**Affiliations:** ^1^ Institute of Microbiology, Technical University Braunschweig Braunschweig Germany

## Abstract

Imposition of restrictions on civil liberties in response to epi/pandemic crises provokes collateral health, economic and social crises. Moreover, as a result of the societal distress engendered, they become less effective over time, reflected in reducing acceptability, public protests, lack of compliance and civil disobedience, as evidenced by current events in some countries. There is an urgent need to evolve new containment strategies that minimize civil liberty restrictions. This requires strategic economic policies to invest in what might be termed *pandemic containment innovation*, particularly in the development of new means of reducing virus concentrations in closed spaces, and of precision exclusion of virus transmitters from public assemblies. Such innovations and their implementation will in turn create significant employment and boost economies. And, because such investments aim at increasing the resilience of society, healthcare and the economy to pandemics (and indeed outbreaks of respiratory infections in general), they are particularly sustainable.

## Restriction of civil liberties comes at enormous health, economic and social costs

Mass imposition of restrictions of civil liberties to contain epi/pandemic outbreaks has been practiced for centuries, most notably during the plague (https://www.wnycstudios.org/podcasts/anthropocene‐reviewed/episodes/anthropocene‐reviewed‐john‐green‐plague) and the Spanish Influenza pandemic (http://traces.web.unc.edu/files/2019/01/v6‐Article‐Ong.pdf). As we have seen in the current COVID‐19 pandemic, lockdown works (Flaxman *et al*., 2020). The question though is: at what cost? Restricting movement of people in response to the COVID‐19 crisis has created collateral health (Sud *et al*., 2020; Timmis, *et al*., 2020), social (https://www.undp.org/content/undp/en/home/coronavirus/socio‐economic‐impact‐of‐covid‐19.html) and economic (https://www.worldbank.org/en/news/feature/2020/06/08/the‐global‐economic‐outlook‐during‐the‐covid‐19‐pandemic‐a‐changed‐world; https://www.bbc.com/news/business‐51706225) crises. These crises create anxiety and suffering in society, which in turn may reduce acceptability and provoke public protests, lack of compliance and civil disobedience. This inexorably leads to a second question, which is: are containment measures developed centuries ago, before it was even known what caused epi/pandemics, with all their collateral damage, still appropriate today? With our vast current knowledge, technology, financial capacity and global networks, we can certainly devise smarter, precision responses to containment that are less disruptive to civil liberties, and hence our health capacities and economies. And, thirdly, the corollary: can we couple the current need to boost economies with job‐creating investments, with the creation of the technical and logistical wherewithal needed to attain and implement such smarter responses?

## Investments to boost economic recovery should target innovation in pandemic containment and healthcare resilience

Governments worldwide are struggling with multiple and interconnected crises caused by COVID‐19: rising infection/disease/death rates, inability of healthcare systems to provide normal services with the ensuing increase in non‐pandemic morbidities and mortalities (Timmis *et al*., 2020), inadequate virus testing capacities, virus containment measures that involve massive restrictions of civil liberties, interruptions of the operation of schools/kindergartens/higher education, home working – often under stressful conditions, economic recession and job losses, rises in stress‐related mental disorders, disruption of social interactions within and among families, lowering of quality of life for large sectors of society, etc. Dealing with these and other issues is a formidable challenge and not made easier by the unpredictable nature of the pandemic.

But, understandably, the economic fall‐out of the pandemic and development of policies and measures to boost economic growth are major preoccupations of policy makers. The key issue here is that the major problems to be addressed are all interconnected, so new policies to deal with the economy need to factor this in. Most importantly, COVID is likely to be with us for a long time, and will continue to negatively impact societies generally, and economies specifically. And, even if/when COVID‐19 is beaten, the next pandemic, which might be even more catastrophic for health and economies, is waiting and may even be just around the corner. To *sustainably* boost economies and create employment, it is essential that governments invest in new innovations that target healthcare resilience to pandemics, especially the reduction of transmission, in order to minimize their impact on health, economies and restrictions of personal liberties.

## Pandemic containment innovation: a sustainable strategy to boost the economy and employment, while increasing societal resilience to pandemics

There are a number of hubs of health innovation activity that could, if adequately incentivized, contribute substantially to pandemic containment and resilience, and to employment creation, but two would appear to be particularly worthy of consideration.

### Virus extraction‐inactivation: innovation in closed space ventilation and air recycling technology

It is now well established that a major mode of transmission of SARS is via aerosols, and that closed spaces with poor ventilation, or closed‐circuit ventilation, are particularly problematic (Morawska *et al*., 2009; Morawska and Milton, 2020). In fact, they are equally problematic for respiratory infections in general and probably responsible for the high incidence of influenza and other winter viral infections. One of the factors in this is the building mantra of energy economy: conserve the heat generated to warm a building. This has increasingly created living, working, education, shopping, places of worship, sport and leisure, medical, travel (hotels, trains, aeroplanes, ferries, etc.), etc., spaces that lack windows which open for fresh air, and have central air circulation systems that recycle air with minimal replenishment of fresh air. Virus‐containing aerosols accumulate in such closed spaces, where they may infect those exposed, and, if circulated, are transmitted to connected spaces where they may infect others. While energy conservation is clearly important, and one of the planks of energy sustainability, the true costs of a pandemic need urgent consideration in the context of policies for our networked endeavours and environment. Energy conservation can no longer be considered in isolation, independently of health needs: energy cost savings need to be viewed in the context of potential cost expenditures resulting from increased transmission of infective agents in closed spaces and the resulting propagation‐perpetuation of outbreaks. And sustainability also requires a functioning society.

The virus concentration in air directly impacts both infection rates and disease severity (Guallar *et al*., 2020; Pujadas *et al*., 2020), so we need modifications of closed spaces that significantly reduce virus concentration. This can be achieved through dilution, by increasing the exchange of stale air with fresh air, and virus removal by filtration and/or inactivation. While it will be relatively straightforward to introduce into new buildings new, effective systems to dilute, remove and inactivate viruses, the problem will be to retrofit effective systems in existing buildings. This is clearly a massive but essential undertaking; existing filtration‐pathogen inactivation systems may not be optimal for purpose. We urgently need innovation in this area: new approaches to filter out or inactivate virus in air, and commercial development of new filtration/inactivation systems. Downstream, there are manufacturing of components and complete systems, sales, installation, servicing, training and recruitment of staff. Given the scale of the problem – the need to modify a major fraction of existing buildings (obviously prioritizing over time) – this type of investment will create both significant new employment, and healthier educational‐living‐working‐care‐leisure spaces that will increase our resilience to current and future pandemics of respiratory infections.

### Transmitter identification and extraction: innovation in diagnostics technology

Current actions to minimize transmission from infectious individuals in communities/regions/countries with rising case incidence rates are to restrict the civil liberties of everyone. For every individual transmitter contained, thousands of non‐spreaders are subjected to draconian civil liberty restrictions which paralyse the normal activities and businesses of entire communities. But, because of the need to allow at least part of society, such as children in schools, etc. to function quasi‐normally, these restrictions still do not isolate important spreaders. If it were possible to target civil liberty restrictions to just those who are infectious, this would have massive life‐ and economy‐changing improvements – in effect allowing society to function almost normally – and represent a policy that everyone can readily understand and accept. Understanding and acceptance are key pre‐requisites for compliance.

If we make the assumption that someone testing negative for SARS‐CoV‐2 will have a low probability of infecting others for several hours^1^, then allowing that person and all others testing negative to engage in normal pursuits would be both beneficial in many ways and represent a tolerable degree of risk. For example, kindergartens, schools, universities, leisure venues like restaurants, bars, gyms, sports venues and many others, could operate normally, without social distancing and possibly without a need for masks.

For a SARS test to function as a *gatekeeper*, it would need to be fast and reliable: while‐you‐wait tests need to provide reliable results within a few minutes. At the moment, tests are coming on stream that can provide results in 15‐30 min (Huang *et al*., 2020), which represents important progress but is not ideal. Tests which give results in 10 min may well be within reach (Wei Huang, pers. comm.). But if we (Kennedy) can decide that a moonshot will be made in the 1960s, we can surely produce a more rapid gatekeeping COVID test in the 21st century! We need to significantly invest in innovation in the development of simple rapid tests suitable for use on queues outside venues. (Of course, the technological advances underpinning such tests will undoubtedly have major benefits for diverse diagnostic applications, including more widespread DIY/home diagnostics, and resulting shortening of diagnostic‐prevention‐treatment pathways leading to improvements in precision medicine and community health.)

While the use of rapid tests for gatekeeping could be used for any members of the population, they would be particularly important for the most problematic segment of society, namely young people, who have become important spreaders and who have the greatest need for the social interactions that are conducive to transmission.

### For example: the 18–30 group

Trying to prevent young people from enjoying themselves in social groups and pairs, including more intimate interactions, is not only virtually impossible but undesirable. Rather than complaining and blaming, we need to energetically explore possibilities for solutions. Here the AIDS epidemic can be instructive. Once young people realized the hazards of unprotected sex, they quickly adopted condom use as the norm, which had a major impact on HIV transmission. At the moment, hazards of COVID‐19 seem almost academic to young people because they generally have mild symptoms. However, examples of more severe cases of COVID‐19 in the young, and infections resulting in unpleasant, sometimes long‐lasting sequelae, are accumulating (Cunningham *et al*., 2020), and it should soon be possible to provide young people with a stark picture of potential severe outcomes of COVID for their age group. If we can couple education campaigns based on this and the obvious hazards young people pose for older generations, with tolerable anti‐transmission measures they can readily accept, we may be able to reduce transmission among young people without imposing unacceptable restrictions on their recreational pursuits.

One measure would be testing at venues young people visit (bars, night clubs, rock concerts, etc.), prior to admission. A negative test should mean that she/he is not at that moment infective; obviously, the person may nevertheless be already infected, and the next day may become infective but, at that moment is not a significant transmission risk, so can enter the venue.^2^ Crucially, anyone testing positive would be immediately quarantined, which would reduce the probability of such venues becoming hot spots of transmission, and thereby support their ability to remain economically viable. Once testing at closed venues became routine, it would be straightforward to extend it to more open venues where organized gatherings (i.e., for which there is an organizer, who would take responsibility for arranging a testing operation) of young people take place.

### For example: the 4–18 group

Everyone agrees that it is essential for children to go to school. The issue is: at what cost? Many teachers are, because of age or underlying conditions, vulnerable to severe outcomes of COVID‐19 infection. Do we simply accept the likely incidence of teacher morbidity and mortality as unavoidable collateral damage? And, as children infect one another and transport coronavirus into their families, who then transmit to vulnerable relatives and friends, including those in care homes, etc., do we simply accept the resulting morbidities and mortalities of family and friends as unavoidable collateral damage? If so, our values and ethics would seem to have undergone a serious deterioration. If not: what to do? If we can test all children (and teachers, auxiliaries, staff) every day at the school gates, we will seriously reduce the risk of transmission in schools, enable schools to function normally without social distancing, and catch the transmitters for isolation before they can do serious damage.

Another important aspect of the restriction of civil liberties is the quarantining of contacts of infected individuals identified by test‐and‐trace programmes, and of those having visited regions with high case incidences. Once again, civil liberty restrictions are imposed on many non‐infected individuals in order to prevent transmission by the few who are infected. The daily testing of test‐and‐trace contacts/travellers from high incidence regions would allow them to continue normal activities, and only those few who subsequently test positive would be obliged to self‐quarantine.

Moreover, the use of virus test gatekeeping can operate just as well in the opposite direction, namely to allow quarantined individuals to return to normal life as soon as they become non‐infectious. Daily testing of quarantined persons would allow precise determination of the time of cessation of virus shedding/infectivity and enable exit from quarantine, in some cases prior to the standard 14‐day period. An early but safe exit from quarantine enabled by test gatekeeping would clearly have significant benefits for everyone, especially for essential workers like medical staff, teachers, and so on.

If testing gatekeeping were to be established as the norm during a pandemic, there would need to be a considerable logistical effort to avoid substantial inconvenience and loss of time for those being tested: there would need to be trained testers in numbers corresponding to a reasonable ratio of testers:tested. But this is simply a logistical issue and would involve employment of large numbers of people. Since this is a public health measure, it could initially be financed by the taxpayer, subsequently by the industry involved. However, since the hospitality industry ranges widely in profitability, some form of risk adjustment‐capitation may be envisaged (Kutzin, 2001; Rice and Smith, 2001).

The development and deployment of rapid tests for gatekeeping purposes would thus involve major innovations in diagnostics and create considerable employment in research, development, manufacturing, sales, human resource training and recruitment, and on‐site use: another example of strategic investment in pandemic management creating significant employment. Virus test gatekeeping should subsequently be automated, but this again would involve employment‐creating innovation.

There are obviously a number of other possible lines of employment creation in innovation aimed at reducing transmission of infectious agents: the construction and equipment of dedicated diagnostic centres (dMCs, see Timmis and Timmis, 2017) that can serve for comprehensive testing (see Timmis *et al*., 2020), development of new anti‐viral disinfectant products and hand sanitizers that are less harmful to skin physiology/microbiomes, development of robots for tasks in high infection risk areas, such as routine surface decontamination (Yang *et al*., 2020).

## Concluding remarks

While current efforts to boost economic activity and maintain some of the existing/create new employment are important and necessary, they should not attempt to simply re‐create the past and invest primarily in traditional activities, some of which unfortunately have a poor outlook. Rather, they need to focus on innovation in areas of the economy that (i) will flourish in the future and (ii) contribute to the resilience of society with regard to this and future pandemics (see also Timmis and Brüssow, 2020). The integration of investment in employment creation with efforts to counter respiratory infections constitutes *one investment, two urgent solutions*.

Current measures to contain COVID‐19, involving major restrictions of civil liberties (Studdert *et al*., 2020) that have enormous negative consequences on economies, livelihoods, mental health and quality of life, fail to specifically target the true problem, namely individuals who are infective. Because in seeking to solve one problem, they entrain other problems that are extremely serious, they are unsustainable. They are exceptionally blunt, temporary measures waiting for an effective vaccine. While they may have been the only option at the beginning of this pandemic, it is evident that we now need new virus containment strategies that specifically target spreaders and that allow life to continue as normal for non‐spreaders. *There is an urgent need for a transformative change*.

Here, I argue that significant new investment must be made in what might be termed *pandemic containment innovation*, particularly in the development of new means of reducing virus concentrations in closed spaces, and virus transmitters in public assemblies. Innovations to reduce virus concentrations in closed spaces are also likely to reduce the amounts of other unhealthy materials in the air, including fungal spores and toxic chemicals, thereby creating healthier indoor spaces. Investment in new means of reducing SARS‐CoV‐2 transmission will also result in massive reductions in transmission of other respiratory pathogens, such as influenza virus, and thus both the primary diseases and any collateral morbidities they cause, so will have significant positive consequences for the health of society in general, including the health cost savings these will entrain and the economic value of the resulting increased societal productivity.

## Endnotes

1 Caveat: this assumption is obviously important to what follows. If examples of a negative test being followed rapidly by high levels of virus shedding are found to occur at significant frequencies, then periods between testing would need to decrease.

2 Of course, nothing in biology is 100%. There will be newly infected individuals that test negative at the gate and that start to shed virus later on in the venue. But this will probably be a rare event and the aerosols created should have low virus concentrations. Nevertheless, intimate interactions involving such a person may result in transmission, but the occasional low, mostly 1:1 transmissions, should have far lower societal impact than current non‐targeted restrictions on civil liberties. Superspreading, resulting from a false‐negative test due to testing failure, should be rare.
